# Medical Opinion and Movement

**Published:** 1909-08-07

**Authors:** 


### Medical Opinion and Movement.

A RESEARCH, extending over three years, into the Enzyme Treatment of Malignant Disease associated with the name of Dr. Beard is published in the *Medical Record* by Dr. Bainbridge. The number of cases included is very large, and the completeness with which the treatment was applied and studied has been monumental; the very greatest care was taken throughout to follow exactly the directions of Dr. Beard, and the results are tabulated and checked with the utmost pains. From the summary of thirty conclusions, which may be taken as the final word on this subject, the following are worth quoting. The enzyme treatment as administered according to the suggestions of Dr. Beard, plus important details of *régime*, does not check the cancerous process; it does not prevent metastasis; it does not cure cancer. The injections are often painful, and many patients refuse to continue them. Because of the tendency of trypsin to disintegrate the tissues it may be a direct menace to life by eroding large blood-vessels or by overwhelming the system with tumour toxins; thus in some cases hastening death. Injectio amylopsini seems in some cases to diminish cachexia, but in others has no such action. Control cases given injections of glycerin and water, or sterile water only, plus the *régime*, did just as well as those on full enzyme treatment. The *régime*, by increasing the general resistance of the patient, may in some cases decrease the rapidity of the malignant process.

THE Clinical and Post-mortem Aspects of the Status Lymphaticus are discussed in the *Journal of Mental Science* by Mr. R. E. Humphry, who believes this condition to be not nearly so rare as many people suppose. He certainly has fairly good grounds for this belief, inasmuch as he has had personal experience of six cases, five of them in asylum patients. The physical signs and symptoms, as described hitherto, are indefinite, but this is because they have been insufficiently investigated. As far as the pathological anatomy of the disease is concerned he notes especially a peculiar pink colour of the enlarged glands, and also an invariable enlargement of the networks of miliary glands in the stomach and intestines. These two signs, with a thymus weighing at least an ounce and multiple glandular swellings everywhere, are diagnostic of the condition. The only other diseases in which a thymus of that size is always found are thymus tod, exophthalmic goitre, and leukæmia; and less constantly in acromegaly, Hodgkin's disease, and myxœdema. The heart muscle is frequently flabby and friable, and fatty degeneration in its walls is common; nevertheless it does not convey the impression that death is due primarily to cardiac failure, and the cavities are nearly always empty; sometimes there is a small quantity of blood in the right auricle. Hypoplasia of the aorta and other arteries is sometimes described as an associated condition. In one of his cases the author found an intense nephritis.

CLINICALLY the symptoms and signs during life are very vague. Deaths from the status lymphaticus are commonest at fifteen to eighteen, though they have been recorded at all ages from one year to fifty. Females are less often affected than males, and an hereditary disposition is said to be proved. The disease is also more common in those of a lymphatic diathesis, and in the offspring of neuropathic stock. The persistent and enlarged thymus seldom, if ever, gives rise to appreciable dulness to percussion of the manubrium: on the contrary, says Mr. Humphry, by pushing the heart further from the chest wall in front, it lowers the upper border of superficial cardiac dulness, and this in the absence of emphysema he regards as rather an important sign of the presence of the thymus. The thyroid gland is often slightly enlarged. The pulse may be less well sustained than normally, and the liver or spleen may be enlarged to a variable extent. But none of these signs is sufficiently constant to be reliable. The cervical and inguinal glands are rarely more palpable than usual. Tonsils and adenoids are frequent; and the so-called pharyngeal and lingual tonsils, and even the uvula may be more or less large. The most important sign of all is the conspicuity of the circumvallate pupillæ of the tongue, and of the mass of lymphoid tissue between them and the epiglottis—this is pathognomonic when it exists, but unfortunately its absence does not exclude the possibility of the disease.

THE ætiology of Bell's Paralysis, or Refrigeratory Facial Paralysis, as he calls it, is considered at length in *International Clinics* by Dr. Reik, of the Johns Hopkins University, who falls foul of the standard text-books in the English language on both sides of the Atlantic for their adherence to the neuritic explanation of this lesion. Most of them imply, he says, that the neuritis is a result of cold upon the trunk or terminals of the nerve; whereas what he regards as the true pathology of the condition is described by many French and German authorities, namely, an antecedent otitis media, not necessarily purulent or obstinate. Statistics are supposed to show that of cases of Bell's palsy some 3 per cent. are due to syphilis, 6 to 9 per cent. to chronic suppurative and necrotic otitis, and 70 to 75 per cent. to cold or rheumatism. His conception of the real origin of these latter cases is that in consequence of lowered local resistance owing to draught, cold, or immersion there is inflammatory swelling of the mucuous membrane and a pouring out of exudate into the tympanic cavity. If there be any break in the facial canal-wall, no matter how small, sufficient to permit the entrance of fluid or of pathogenic bacteria, there exists the opportunity for a direct extension of the inflammation to the nerve, or for its compression within its confined channel. It follows that treatment should always be directed to the middle ear in these patients; and the author describes cases in which an apparently normal drum has been incised for this reason and found to contain serum or bloodclot. He claims that in refrigeratory paralysis there is always to be obtained by diligent search some history of antecedent earache or some slight impairment of the auditory sense, and recommends paracentesis as a routine whether this is so or not. The results on the paralysis of such treatment are reported to be excellent.

---

AT a recent meeting of the Société de Biologie, Nicolaïdi gave an account of his researches into the physiological action of certain Solutions of Mineral Salts and Acids, and Radio-active Solutions, when injected into animals. Fluid containing phosphoric acid and glycero-phosphates was found to produce an increase in the nutritive exchanges and body weight when introduced into rabbits. Similar results were obtained with progressively increasing doses of phosphoric acid injected into a dog and other rabbits. Inspired by the results of his earlier work on radio-active bodies, the author next tried the effect of using similar solutions, which he made radio-active by the method of Jaboin. A more marked increase in body weight and in the nutritive exchanges, as well as an augmentation in the number of the blood-cells, were found after employing these radio-active solutions, and no macroscopic or microscopic changes were subsequently discovered in the organs. These results induced the author to employ this form of treatment in the human subject, and in the course of a year he has performed some 700 injections with favourable results in cases of cachexia, malnutrition, and certain infectious diseases. These favourable effects would seem to have been caused by the injections modifying the nature of the "soil," and re-establishing a condition of nutritional equilibrium.

---

A SOMEWHAT rare complication of Mumps, yet one which has been met with several times in the course of particular epidemics, is described by Paul Roy in a recent number of *La Clinique*. At any time between the first and twelfth days of the disease, usually from the third to the sixth, the patient is attacked with severe pain in the epigastrium, accompanied by nausea and vomiting, and is occasionally found to have a palpable tender tumour in a situation corresponding to the pancreas. A careful examination will exclude the possibility of the presence of an appendicitis, gall-stones, perforated gastric ulcer, and the gastric crises of tabes. The complication is in reality an extension of the infectious process to the gland cells of the pancreas, analogous to a similar spread which occurs at times to the ovaries and testicles. It is usually met with in young adolescents, and has been noticed particularly in young soldiers. There would seem to be no connection between the severity of the attack and the onset of pancreatitis. Nor does this complication seem to have any influence on, nor to be influenced by, other complications. The cause of its incidence, as well as the origin of the pain and digestive disturbance, are obscure, but the latter are usually attributed to pancreatic congestion and irritation of the solar plexus.

PAIN is present in most of these cases, but may only be elicited by pressure. At first it is dull and deep seated, but gradually increases in severity, and may ultimately even lead to syncope. It is usually paroxysmal, and seated in the epigastrium, midway between the umbilicus and the ensiform process. It may radiate to the back or along the sides towards the floating ribs. Vomiting is usually present, and varies in intensity from simple nausea to intractable vomiting. The first vomit contains food, later is watery and bilious, and is occasionally hæmorrhagic. Constipation may be present, but diarrhœa is more common and often excessive. Jaundice, contrary to what might be expected, is very rare. Fever, which is proportional to the severity of the pancreatitis, varies from 100° to 102° F., and may last three or four days. Local and functional symptoms are very rare, palpation usually revealing no appreciable increase in size of the organ. Albuminuria is rarely present, and there is never glycosuria or steatorrhœa. The course of the complication is rapid, lasting from two to seven days. Fever is the first to disappear, then vomiting, and lastly pain, which lasts longest of all symptoms. A complete cure is the usual result, and it would seem that no permanent organic injury remains. Diagnosis may be difficult, especially when the ordinary symptoms of mumps are suppressed. Treatment is purely symptomatic, and is directed chiefly to relieving the pain and vomiting. Ice applied to the epigastrium and allowed to melt in the mouth, mixtures containing opium and cocaine and hypodermic injections of morphia are useful to this end.

---

IN a recent number of the *Revue de Stomatologie* attention is called by Chompret to the advantages of performing Alveolar Syndesmotomy, or section of the alveolo-dental ligaments, prior to extracting teeth. The circular ligament is divided, and, after penetrating more or less deeply into the joint itself, the fibres of the alveolo-dental ligament are severed. To effect this variously shaped slender instruments of fine temper and exceedingly sharp are necessary. The advantages claimed for this operation are the certainty with which tearing of the gum is avoided and the adherence of the tooth diminished, and the increased facility in getting a grip of the tooth with the elevator or forceps. Moreover, the shape of the roots can easily be determined, so that this preliminary operation facilitates subsequent extraction in difficult cases. The operation can be performed without pain under a local anæsthetic, and the additional time necessitated is more than counterbalanced by the resulting advantages.

---

AT the last meeting of the Biological Society of Paris, Dr. A. Netter referred again to the administration of Chloride of Calcium as a preventive means against cutaneous eruptions following Injections of Serum, as he does not think its efficacy in this respect is sufficiently well realised by the profession. He gives some interesting statistics to substantiate his point. In 1907 among 229

diphtheritic patients who received serum injection, and were also treated with chloride of calcium, only 7—that is, 3.1 per cent.—developed a rash, whereas of 251 diphtheritic patients who did not have the salt, 43—or 17.1 per cent.—had an eruption. During the first six months of the present year there have been five cases of a rash in 117 cases of diphtheria with chloride of calcium—that is, 4.3 per cent.—and 25 cases of a rash in 133 cases without the salt, or 18.8 per cent. The administration of chloride of calcium should commence directly the first injection of serum is made, and should continue for three or four days, or longer if the injections are repeated. The author states that the preventive action of the salt is much less efficacious for the intra-spinal injections of anti meningococcal serum. He is unable to offer any satisfactory explanation of the action of the salt, but the statistics he furnishes favour strongly the use of the drug in these cases, and they are sufficiently large to exclude the element of chance.

---

MOST ingenious are the means sometimes adopted to simulate disease in order to avoid military service on the Continent. Some cases of tumours in the legs, produced by the subcutaneous injection of paraffin wax, were recently reported in these columns. The latest form of simulation for this purpose is the production of an apparent jaundice by the ingestion of picric acid in gelatin capsules. Three cases are reported from the Odessa military hospital. Thirty-centigramme doses were taken twice a week. From the third day the skin and sclerotics became saffron yellow, and the absorption of six to eight doses were sufficient to make the colour persist for more than three weeks afterwards. The surgeon, Dr. Pievnitsky, was able to arrive at a correct diagnosis from the fact that the usual attending symptoms of true jaundice were absent. The pulse was rather accelerated than slow (in one case as much as 100 per minute), the appetite and sleep were not disturbed, the fæces were of a normal colour, and there was a tendency to diarrhœa. The urine, sometimes red and sometimes blackish, contained neither biliary acids nor pigments. Treated with sulphuric acid it became rapidly decolourised, so that this condition of the urine, and especially the absence of bilirubin, were sufficient in themselves to determine the nature of the condition.

---

THERE are many so-called diagnostic signs of pathological conditions such as aortic insufficiency, which are by no means constant, often only appear when the condition is well-established, and are consequently of little diagnostic value, though nevertheless interesting pathological phenomena. Among such may be classed the "Circulatory Pupil" recently described by Dr. Michel Landolfi of the Naples Incurable Hospital. It appears that previously in 1903, Dr Roche of Geneva conceived the idea that in aortic insufficiency the pupil might show rhythmic variations corresponding to the cardiac pulsations, but careful examination of several patients failed to confirm this supposition. In one case, however, it is reported that the iris showed variations synchronous with the respiration. Dr. Landolfi following up the same idea has been more successful and has actually observed this "circulatory pupil" in one case among twenty-four patients examined, all exhibiting the classic symptoms of aortic insufficiency. By the administration however of digitalis for three or four days he was able to obtain the same phenomena in three other cases. The rhythmic variations of the pupil are, of course, independent of light, the contraction of the pupil corresponding to ventricular systole dilatation to diastole. This phenomena is apparently only present in cases of pure aortic insufficiency, not complicated with aneurysm, aortic stenosis or other valvular lesions, and when there is well-marked hypertrophy of the left ventricle and a Corrigin pulse. The explanation of the phenomenon is that during systole the rush of blood causes hyperæmia of the iris and consequently myosis; during diastole the rapid return of the blood induces anæmia of the iris and so mydriasis. It will be interesting to note whether this phenomenon receives further confirmation at the hands of other observers.

---

THE application of the Galvanic Current in cases of Facial Neuralgia too often fails to effect a satisfactory cure or even to give any substantial relief from the affliction. Dr. V. Vitck of Prague had recourse to galvanisation in a case of severe neuralgia of the second and third branches of the Trigeminal, applying the positive pole in the usual manner at the level of the suborbital and mental foramina. Although treatment was carried out twice a day little relief was obtained, the attacks became less frequent, but of the same intensity. The idea then occurred to Dr. Vitck to place the electrode inside the mouth at the level of the suborbital foramen and also on the inner side of the inferior maxilla near the temporo-maxillary articulation where the inferior dental nerve passes at the side of the lingual before entering the dental canal. The current was applied at these points for five minutes beginning with a strength of one milliampere and gradually increasing. The effect was, according to the author surprising. At the end of three days the patient was able to eat and sleep without pain, the attacks of pain became more and more rare, and at the end of a week the neuralgia had completely disappeared. Since then Dr. Vitck has had the same success in two other similar cases, and he explains these good results by the fact that in this way the current comes into closer contact with the affected nerves. He has had a special electrode constructed for the purpose, consisting of a long handle insulated with hardened gutta percha and terminating in a sort of short, slightly curved spatula. Dr. Vitck has also successfully treated a case of supra-orbital neuralgia by placing the electrode under the eyelid in the position of the supra-orbital foramen. Care must be taken to only use a very weak current (0.5 milliampere at the most), and not to leave the electrode in contact with the conjunctiva for more than a minute at a time.

